# Multimodal and systemic therapy with cabozantinib for treatment of recurrent hepatocellular carcinoma after liver transplantation: A case report with long term follow-up outcomes

**DOI:** 10.1097/MD.0000000000027082

**Published:** 2021-09-24

**Authors:** Robert Mahn, Farsaneh Sadeghlar, Alexandra Bartels, Taotao Zhou, Tobias Weismüller, Patrick Kupczyk, Carsten Meyer, Florian C. Gaertner, Marieta Toma, Tim Vilz, Petra Knipper, Tim Glowka, Steffen Manekeller, Jörg Kalff, Christian P. Strassburg, Maria A. Gonzalez-Carmona

**Affiliations:** a Department of Internal Medicine I, University Hospital Bonn, Germany; b Department of Radiology, University Hospital Bonn, Germany; c Department of Nuclear Medicine, University Hospital Bonn, Germany; d Department of Pathology, University Hospital Bonn, Germany; e Department of Visceral Surgery, University Hospital Bonn, Germany.

**Keywords:** cabozantinib, living-donor liver transplantation, long-term survival, multimodal therapy, non-cirrhotic HCC

## Abstract

**Rationale:**

Recurrence of hepatocellular carcinoma (HCC) after liver transplantation (LT) remains a major therapeutic challenge. In recent years, new molecular-targeted therapies, such as cabozantinib, have been approved for the treatment of advanced HCC. However, clinical experience with these new drugs in the treatment of HCC in the LT setting is very limited.

**Patient concerns:**

In 2003, a 36-year-old woman was referred to the hospital with right upper abdominal pain.

**Diagnosis:**

An initial ultrasound of the liver demonstrated a large unclear lesion of the left lobe of the liver. The magnet resonance imaging findings confirmed a multifocal inoperable HCC in a non-cirrhotic liver. Seven years after receiving a living donor LT, pulmonary and intra-hepatic recurrence of the HCC was radiologically diagnosed and histologically confirmed.

**Interventions:**

Following an interdisciplinary therapy concept consisting of surgical, interventional-radiological (with radiofrequency ablation [RFA]) as well as systemic treatment, the patient achieved a survival of more than 10 years after tumor recurrence. As systemic first line therapy with sorafenib was accompanied by grade 3 to 4 toxicities, such as mucositis, hand-foot skin reaction, diarrhea, liver dysfunction, and hyperthyroidism, it had to be discontinued. After switching to cabozantinib from June 2018 to April 2020, partial remission of all tumor manifestations was achieved. The treatment of the remaining liver metastasis could be completed by RFA. The therapy with cabozantinib was well tolerated, only mild arterial hypertension and grade 1 to 2 mucositis were observed. Liver transplant function was stable during the therapy, no drug interaction with immunosuppressive drugs was observed.

**Outcomes:**

More than 10 years survival after recurrence of HCC after living-donor LT due to intensive multimodal therapy concepts, including surgery, RFA, and systemic therapy with cabozantinib in the second line therapy.

**Lessons:**

In conclusion, this report highlights the tolerability and effectiveness of cabozantinib for the treatment of HCC recurrence after LT. We show that our patient with a late recurrence of HCC after LT benefitted from intensive multimodal therapy concepts, including surgery, RFA, and systemic therapy.

## 1 Introduction

Hepatocellular carcinoma (HCC) is the most common primary liver cancer worldwide.^[1]^ Liver cirrhosis represents a major risk factor for HCC development, and HCC is the first cause of death in patients with liver cirrhosis.^[2]^ HCC arising in a non-cirrhotic (NC-HCC) or non-fibrotic liver is a fairly rare malignancy. NC-HCC often affects younger healthy female patients and it is usually larger at diagnosis, since the patients did not participate in HCC surveillance programs. In these patients, surgical resection with curative intent is the treatment of choice.^[3–5]^ For HCC in a cirrhotic liver within the Milan criteria (MC) (a single tumor of up to 5 cm or up to 3 tumors, each no larger than 3 cm) without macrovascular or extra-hepatic manifestations, liver transplantation (LT) represents the best treatment option with 5-year survival rates of more than 70%.^[6–8]^ Conventional LT for HCC beyond MC for selected patients may also achieve acceptable overall survival (OS) and recurrence rates.^[9,10]^ Due to deceased organ donation shortage, the use of living-donor liver transplantation (LDLT) is generally accepted for patients beyond MC. However, while LT for patients with unresectable NC-HCC out of MC is not well established, it seems to achieve 5-year survival rates of up to 59%.^[11]^

HCC recurrence after LT remains a major therapeutic challenge and is of limiting prognosis.^[12,13]^ In most cases, recurrence occurs within the first 2 years. However, cases of very late recurrence have also been reported.^[14]^ For patients with oligometastatic intra-hepatic or extra-hepatic tumor recurrence, surgical or interventional-radiological treatments may be applied with curative intention.^[12,13,15]^ In cases of unresectable, recurrent HCC, a systemic treatment is recommended. From 2007 to 2015, sorafenib, a multikinase inhibitor, was the only therapeutic agent showing a survival benefit in patients with advanced HCC compared to placebo.^[16]^ In recent years, new molecular-targeted therapies, such as lenvatinib, regorafenib, cabozantinib, and ramucirumab, as well as immune checkpoint inhibitors, such as nivolumab, pembrolizumab, and atezolizumab in combination with bevacizumab, have been approved for the treatment of advanced HCC.^[17–21]^ However, clinical experience with these new drugs in the treatment of HCC after LT is very limited, since these patients are excluded from phase III trials. Some evidence can be found for sorafenib in combination with rapamycin inhibitors for recurrent HCC after LT and for regorafenib as systemic second line therapy.^[22–24]^

Here, we report on efficacy, feasibility, and tolerability of cabozantinib in a female patient with recurrent HCC after LDLT and who was intolerant to sorafenib. Moreover, we show the benefits from intensive multimodal therapy concepts, including surgery, radiofrequency ablation (RFA), and systemic therapy resulting in a long-term survival of more than 10 years after HCC recurrence.

## 2 Case presentation

**Figure 1:**
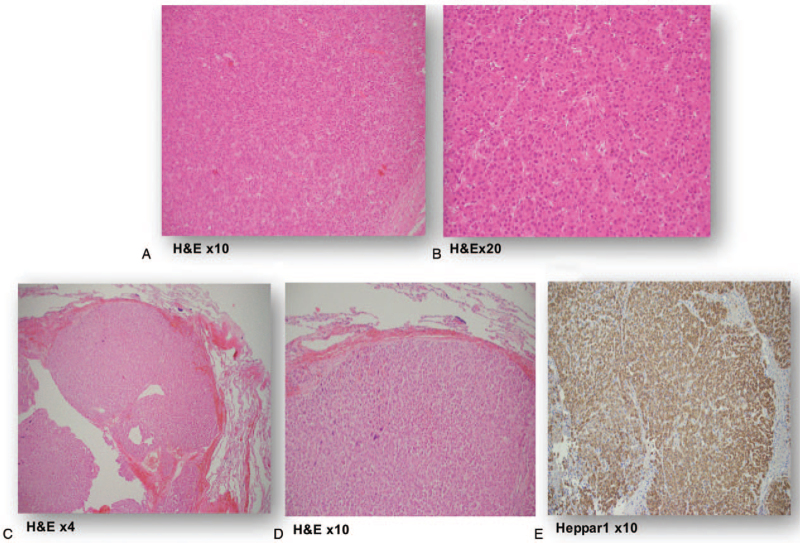
(A/B) H&E staining of partial liver resection with a 9.5 cm, moderately differentiated, hepatocellular carcinoma and multiple intra-hepatic metastases in 10× (A) and 20× magnification (B). Liver parenchyma without cirrhotic changes (C/D/E) H&E and Hepar1 staining from a left thoracotomy with resection of pulmonary metastases of a moderately differentiated hepatocellular carcinoma in 4× (C), 10× (D), and 10× (E) magnification.

**Figure 2:**
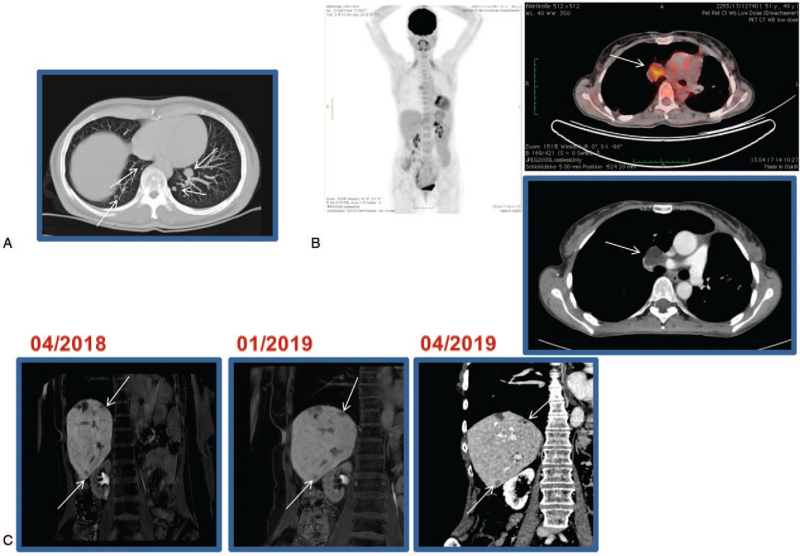
(A) CT scan of thorax dated February 1, 2010 (lung window, axial maximum intensity projection) shows high level suspicion of at least 7 lung metastases on both sides. (B) FDG-PET/CT scan dated April 13, 2017 shows moderately intensive FDG image of the known right hilar lymph node metastasis and CT scan of thorax/abdomen from February 1, 2017 shows progressive size right hilar lymph node metastasis. (C) CT scan of abdomen and MRI of liver from April 2018, January and April 2019 show constancy or low regression of HCC-suspicious lesions in liver segments VI and VIII. **The arrows point to metastases**. HCC = hepatocellular carcinoma, MRI = magnet resonance imaging.

**Figure 3:**
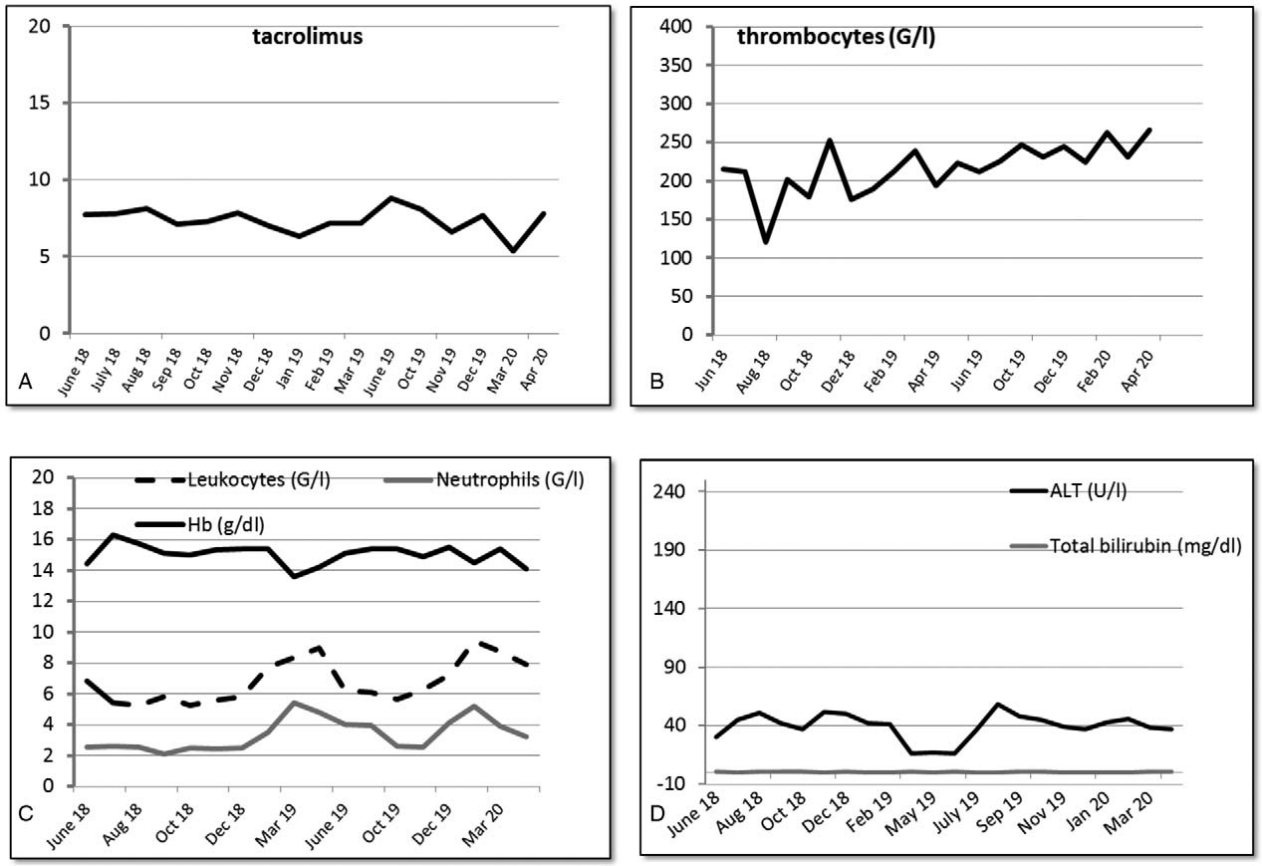
Diagram of the tacrolimus level (A), blood count cells (B/C), total serum bilirubin and ALT levels (D) from June 2018 to April 2020 of the patient during cabozantinib therapy.

In 2003, a 36-year-old Caucasian woman was referred to the hospital with right upper abdominal pain. An initial ultrasound of the liver demonstrated a large unclear lesion of the left lobe of the liver. Serum level of y-GT was slightly elevated at 84 U/mL. The AFP value was negative. Medical history of the patient revealed a slight mitral valve prolapse, latent hypothyroidism, a condition after reconstructive burn surgery in 1969 and previous nicotine consumption. There was no evidence for any underlying liver disease, including viral hepatitis, non-alcoholic fatty liver, autoimmune pathologies, or genetic metabolic disorders. No intakes of alcohol, hepatotoxic drugs, or hormone substitution and no contact with hepatotoxins were documented. Magnet resonance imaging (MRI) findings were consistent with a liver adenoma. However, a highly differentiated HCC could not be ruled out. According to the decision of our interdisciplinary tumor board and after excluding extra-hepatic tumor manifestations (colonoscopy, gastroscopy, CT scan, and whole body FDG-PET/CT scan), an explorative laparotomy with the intent of a left hepatectomy was performed. Intra-operatively, an approx. 9.5 cm large tumor of the left liver lobe was resected. Histopathologic specimen confirmed a moderately differentiated HCC in a NC liver (Fig. 1A/B). However, intra-operatively performed ultrasound detected irresectable multifocal metastases in the right liver. In December 2003, as part of an individual therapy concept, the patient received an orthotopic LDLT of the right hepatic lobe from her mother. LDLT was approved by the ethics committee of the Medical Chamber of North Rhine-Westphalia, Germany. The liver explant contained up to further 7 lesions of a highly differentiated HCC. The definitive tumor stage of the explanted liver was pT3 pN0 (0/4) M0 with no microvascular involvement. Immunosuppression was initially started with tacrolimus and mycophenolate mofetil. In the following time period, the patient showed an excellent graft function. CT and MRI scans were carried out at intervals of about 3 to 6 months as follow-up. Seven years later, in February 2010, CT scan revealed lung metastases (Fig. 2A). Further tumor manifestations were ruled out with FDG-PET/CT scan and MRI of the liver. Immunosuppression was switched to sirolimus. According to the decision of our interdisciplinary tumor board, resection of the pulmonary lesions with curative intention was performed. In February 2010, May 2010, October 2013, and November 2013, further partial lung resections of new lung metastases were performed. Histologically, metastases of the previously known HCC were confirmed (Fig. 1C/D/E). In January 2014, an atypical liver resection was performed due to intra-hepatic HCC recurrence. Pulmonary segment resections followed again in July 2014, November 2014, and August 2015. Because of a new intra-hepatic HCC recurrence in January 2015, RFA of 2 liver metastases was performed. After yet another tumor recurrence in the lung with confirmation of HCC cells in the cytology by bronchoscopy in December 2015, our tumor board decided to start palliative first line systemic therapy with sorafenib. Sorafenib therapy with 800 mg per day was accompanied by grade 3 hand-foot syndrome and pronounced head pruritus. Even after dose reduction to initially 600 mg after 1 month and later 400 mg per day, sorafenib had to be discontinued in February 2016 due to intolerable grade 3 to 4 side effects, such as mucositis, hand-foot skin reaction, diarrhea, liver dysfunction with transaminase elevation, and hyperthyroidism, requiring hospitalization of the patient. Several weeks after the discontinuation of sorafenib and switching the immunosuppression to tacrolimus again, the patient recovered from the side effects. However, grade 1 hyperthyroidism and hand-foot skin reaction persisted. An initial second line therapy with regorafenib was contraindicated, since the patient did not tolerate sorafenib. In February 2017, a further progression of the HCC showing a mediastinal lymph node was documented by CT scan and confirmed by FDG-PET/CT scan (Fig. 2B). In April 2017, due to a lack of further systemic therapeutic options, it was decided to perform right lateral resection of the mediastinal lymph node metastases. The histology again confirmed HCC spreading. Further solitary pulmonary recurrences of HCC could be treated locally by CT-guided RFA in October 2017 and November 2017. In April 2018, MRI scan of the liver revealed newly appeared high-grade HCC-suspected lesions subcapsular in liver segment 8 (10 × 7 mm) and at the segment border 6/7 (7 × 6 mm). After tumor board discussion, a switch to an off label second line systemic therapy with cabozantinib was recommended. Thus, from June 2018 to April 2019, our patient received second line therapy with cabozantinib. An initial start with 40 mg was accompanied by mild (grade 1–2) oropharyngeal mucositis and mild hypertension, both of which were easy to manage. Therefore, the dose could be increased to 60 mg per day with acceptable tolerability. Occasionally, due to mild mucositis and abdominal distension, cabozantinib had to be temporarily reduced to 40 or 20 mg. After 11 months of therapy with cabozantinib, the CT scan revealed complete remission of the pulmonary lesions and low partial remission of the liver metastases as a best response to cabozantinib. After discussion of the case in our interdisciplinary tumor conference, a new round of RFA of the remaining liver metastasis could be completed in April 2019, following discontinuation of cabozantinib. Unfortunately, 2 months later, new progression of the pulmonary metastasis was observed and the therapy with cabozantinib had to be restarted in August 2019. Again, partial remission of all tumor manifestations in the lung was achieved and the therapy with cabozantinib was well tolerated with only mild arterial hypertension, grade 1 to 2 mucositis and gastrointestinal symptoms, all of which could be easily managed. In the further course of therapy, the initial dose of 40 mg/day could be increased to 60 mg/day, while the dose was adjusted to 20 to 60 mg to control the gastrointestinal side effects and mucositis. Liver transplant function and blood count cells remained stable during therapy and no drug interaction with immunosuppressive drugs was observed at any time (Fig. 3A/B/C/D).

## 3 Discussion

Here, we report on efficacy, feasibility, and tolerability of cabozantinib in a female patient intolerant to sorafenib with late recurrent HCC after LDLT due to NC-HCC. Furthermore, we report on the benefits of intensive multimodal therapies, including surgery, RFA, and systemic therapy, achieving long-term survival of more than 10 years after HCC recurrence.

Little is known about the etiology of the HCC in the presented case. The peculiarities of this case were the female sex, the young age, normal body mass index, and the absence of any underlying liver pathology. Whether environmental conditions, such as the inhalation of toxic gases after a fire accident in childhood, and chronic nicotine smoke inhalation played a role as possible triggers for the development of HCC remains unclear.

The first and unique symptom of the patient was right upper abdominal pain. As is common for NC-HCC and due to lack of symptoms, no HCC surveillance or earlier imaging were performed. Therefore, the initial ultrasound of the liver showed an already large tumor at diagnosis, indicating advanced stage.^[5,25–26]^ Here, despite a larger tumor burden, surgical resection is the treatment of choice for HCC in NC liver, resulting in better benefit on survival than HCC in cirrhotic liver.^[3,5]^ Patients with cirrhosis and small, unresectable HCCs within the MC (a single tumor of up to 5 cm or up to 3 tumors, each no larger than 3 cm) without vascular or extra-hepatic manifestations, who undergo orthotopic LT achieve 5-year survival rates of more than 70%.^[6–8]^ LT for patients with unresectable NC-HCC out of MC is not well established. However, as it was the case with our patient, LT may be offered for selected patients showing acceptable survival and recurrence rates.^[9–11]^ A European multicentric analysis reported a 5-year survival of 59% for patients independent of tumor size without macrovascular invasion and/or positive lymph nodes.^[11]^ Because of limited organ supply, LDLT represents an alternative option to deceased donor liver transplantation. The indication for LDLT without waitlist time, as in the case of our patient, provides a curative option to selected patients with irresectable HCC, since HCC progression is limited in time.^[27,28]^ In the case of our patient, a 7-year recurrence-free survival could be achieved.

However, the risk of HCC recurrence after transplantation remains between 8% and 20%.^[29]^ HCC recurrence mostly appears in the first 2 years after LT, but later relapses, as seen in our patient, have also been reported in the literature.^[14]^ The recurrence pattern of these late recurrences often manifests as single extra-hepatic metastases. In these cases, intensive surgical treatment has shown to improve survival. In a large single-center study by Bodzin et al,^[30]^ 106 patients who developed recurrent HCC post-LT were analyzed. Patients who underwent surgical therapy showed an improved median survival of 27.8 months versus 10.6 months for those with non-surgical therapy or 3.7 months for those following a best supportive care concept. Further studies confirmed that surgically accessible HCC recurrence after LT is associated with prolonged OS.^[31,32]^ In addition to surgery, multimodal combined treatment with local and systemic therapies may also result in increased survival, even in patients with HCC metastasis in multiple organs, as was the case in our patient.^[12,13,15]^ Therefore, we assume that the long survival of our patient was due to the initial aggressive surgical and interventional-radiologic treatments until the start of systemic therapy with sorafenib. Furthermore, late recurrences appear to have significant better OS compared to early recurrences within the first 2 years, as they show less aggressive tumor biology and higher response rates to local treatments.^[14]^

When surgical or local therapies are not possible, systemic therapy with sorafenib is usually the recommended first line therapy.^[33]^ Sorafenib was the first drug approved for systemic treatment of advanced HCC.^[16]^ For the use of sorafenib in recurrent HCC after LT, a potential therapeutic benefit has also been shown in several studies, especially in combination with mammalian target of rapamycin inhibitors.^[22,23,33–35]^ In our case, the patient was treated systemically with sorafenib. Immunosuppression was switched to a mammalian target of rapamycin inhibitors (sirolimus). However, the therapy with sorafenib was accompanied by grade 3 to 4 side effects, including mucositis, hand-foot skin reaction, diarrhea, liver dysfunction with transaminase elevation, and hyperthyroidism, requiring hospitalization of the patient and discontinuation of sorafenib. Similarly, in the study by Staufer et al,^[36]^ severe toxicities, including acute hepatitis, diarrhea, hand-foot-skin reaction, and bone marrow suppression were reported in more than 90% of all patients receiving sorafenib with recurrent HCC after LT, indicating a high toxicity of sorafenib in this setting.

Regorafenib was the initial second line systemic treatment approved in patients with HCC. In the phase III RESORCE trial, Bruix et al^[19]^ reported a survival benefit of regorafenib versus placebo as second line therapy in patients who tolerated and progressed under sorafenib. The data on second line therapies after sorafenib failure in posttransplant patients are still poor. A multicentric, retrospective study by Lavarone et al^[24]^ describes the feasibility of regorafenib in posttransplant patients with recurrent HCC who progressed on sorafenib (median OS of 12.9 months after regorafenib initiation and 38.4 months after initiation of sorafenib, with a 1- and 3-year OS of 68% and 23%, respectively). However, due to the poor tolerability of sorafenib, therapy switch to regorafenib was not possible in our patient. Therefore, we opted for systemic second line therapy with cabozantinib. Cabozantinib, a tyrosine kinase inhibitor targeting Vascular Endothelial Growth Factor-Rezeptor 2, AXL, and MET in patients with advanced HCC who progressed on sorafenib was evaluated in the phase III CELESTIAL trial. Compared to placebo, cabozantinib showed a significant benefit in median OS (10.2 vs. 8 months; *P* = .004) and PFS (5.2 vs 1.9 months; *P* < .0001).^[20]^ Due to the lack of clinical experience with cabozantinib in combination with immunosuppressive agents and the potentially increased risk of side effects, we started with great caution at a reduced dose and were able to increase the dose to 60 mg per day in the further treatment course. Despite similar adverse events to sorafenib as described for cabozantinib in the CELESTIAL trial, such as hand-foot syndrome (17%), hypertension (16%), elevated aspartate aminotransferase levels (12%), diarrhea (10%), and fatigue (10%), our patient tolerated the systemic therapy with cabozantinib well.^[20]^ Only grade 1 to 2 toxicities, such as hypertension, mucositis, or gastrointestinal complaints were observed, and no grade 3 or grade 4 toxicities occurred over the long exposition period of more than 20 months. The side effects could be easily managed with occasional dose adjustments as necessary. Furthermore, graft function and the level of tacrolimus remained stable during the entire treatment with cabozantinib and no interactions with immunosuppression were observed at any time. Finally, despite several dose adjustments, an objective response could be achieved with complete remission of the lung metastasis and a low partial remission of the liver metastasis as best response. Moreover, a new round of RFA of the remaining liver metastasis could be completed 11 months later, resulting in complete HCC remission, allowing a temporary discontinuation of the systemic therapy with cabozantinib.

## 4 Conclusion

In conclusion, we report on a patient with inoperable HCC in a NC liver out of MC, who benefitted from LDLT. After suffering a late recurrence of HCC, our patient benefitted from intensive multimodal therapy concepts, including surgery, RFA, and systemic therapy, achieving a long-term survival of more than 10 years. Furthermore, our report highlights feasibility, tolerability, and effectiveness of cabozantinib as the second line therapy for the treatment of HCC recurrence after LT in a patient intolerant to sorafenib.

## Author contributions

MAG made substantial contributions for the conception and supervision of the manuscript.

RM and FS has been involved in drafting and writing the manuscript.

RM, AB, TZ, and TW has been involved in revising the manuscript critically for important intellectual content.

TV, PK, TG, SM, JK, and CPS made substantial contributions for acquisition of clinical data and gave final approval for the version of the manuscript to be published.

CM made substantial contributions for acquisition of radiological data.

MT was involved in the histopathological examinations and their evaluation.

All the authors have made significant contributions to the manuscript and have also read and approved the manuscript.

**Conceptualization:** Maria A. Gonzalez-Carmona.

**Data curation:** Robert Mahn, Farsaneh Sadeghlar, Alexandra Bartels, Taotao Zhou, Tobias Weismüller, Patrick Kupczyk, Carsten Meyer, Marieta Toma, Petra Knipper, Tim Glowka, Steffen Manekeller.

**Formal analysis:** Robert Mahn, Farsaneh Sadeghlar, Patrick Kupczyk.

**Methodology:** Patrick Kupczyk, Carsten Meyer.

**Resources:** Maria A. Gonzalez-Carmona.

**Software:** Carsten Meyer, Florian C. Gaertner.

**Supervision:** Maria A. Gonzalez-Carmona.

**Validation:** Maria A. Gonzalez-Carmona.

**Writing – original draft:** Robert Mahn, Farsaneh Sadeghlar, Patrick Kupczyk.

**Writing – review & editing:** Alexandra Bartels, Taotao Zhou, Tobias Weismüller, Carsten Meyer, Florian C. Gaertner, Marieta Toma, Tim Vilz, Petra Knipper, Tim Glowka, Steffen Manekeller, Jörg Kalff, Christian P. Strassburg, Maria A. Gonzalez-Carmona.
